# The multiple roles of β-diversity help untangle community assembly processes affecting recovery of temperate rocky shores

**DOI:** 10.1098/rsos.171700

**Published:** 2018-08-08

**Authors:** Mariachiara Chiantore, Simon F. Thrush, Valentina Asnaghi, Judi E Hewitt

**Affiliations:** 1 DiSTAV, Università di Genova, Corso Europa, 26, Genoa 16132, Italy; 2 Institute of Marine Science, The University of Auckland, Private Bag 92091, Auckland 1142, New Zealand; 3 National Institute of Water and Atmospheric Research, PO Box 11-115, Hillcrest, Hamilton, New Zealand

**Keywords:** disturbance-recovery, biogenic habitats, ecological connectivity, environmental filters, macroalgae and turf fauna, Italy

## Abstract

Metacommunity theory highlights the potential of β-diversity as a useful link to empirical research, especially in diverse systems where species exhibit a range of stage-dependent dispersal characteristics. To investigate the importance of different components and scales of β-diversity in community assembly, we conducted a large-scale disturbance experiment and compared relative recovery across multiple sites and among plots within sites on the rocky shore. Six sites were spread along 80 km of coastline and, at each site, five plots were established, matching disturbed and undisturbed quadrats. Recovery was not complete at any of the sites after 1 year for either epibenthos (mostly composed of macroalgae and, locally, mussels) or infauna. Significant differences in recovery among sites were observed for epibenthos but not for infauna, suggesting that different community assembly processes were operating. This was supported by epibenthos in the recovering plots having higher species turnover than in undisturbed sediment, and recovery well predicted by local diversity, while infaunal recovery was strongly influenced by the epibenthic community's habitat complexity. However, infaunal community recovery did not simply track formation of habitat by recovering epibenthos, but appeared to be overlain by within-site and among-site aspects of infaunal β-diversity. These results suggest that documenting changes in the large plants and animals alone will be a poor surrogate for rocky shore community assembly processes. No role for ecological connectivity (negative effect of among-site β-diversity) in driving recovery was observed, suggesting a low risk of effects from multiple disturbances propagating along the coast, but a limited resilience at the site scale to large-scale disturbances such as landslides or oil spills.

## 1. Introduction

Despite their theoretical and empirical application to ecology, the relationships between biodiversity and resilience of communities have been hard to define [1–3]. This complexity is inevitable given the multiple aspects of biodiversity, the multiple scales of variability and the quality of empirical data available to test hypothesis and define relationships within and across very different ecological systems [4]. β-diversity is one element of diversity subjected to extensive pattern analysis, particularly with the rise of the concept of metacommunities [5]. Despite debate over the pros and cons of different β-diversity measures [6], theory continues to advance, although applications to better understand the dynamics of natural ecosystems are less common [7–9]. Nevertheless, empirical estimates of β-diversity, along with various forms of landscape connectivity, have been informative in explaining differences in the rates of community recovery [1,10] and the relative importance of regional- versus local-scale recovery processes [11].

β-diversity is a measure of the compositional differences among samples that links local (α-diversity) to regional (*γ*) diversity. β-diversity can be decomposed to reflect the importance of different processes affecting community assembly: *species replacement* (turnover of species as a consequence of environmental sorting or spatial or historic constraints), *richness difference* (one community has more uniquely held species than another), and *nestedness* (the species present at a site with low numbers of species are a subset of the species present at richer sites) [12–14]. Large survey datasets, potentially encompassing a wide range of spatial scales, have been used to disentangle the drivers of β-diversity, but the strong inference associated with manipulative field experiments is often missing. In an experimental context, manipulation or control of potential drivers or successional states can explicitly assess processes and interactions, especially in a disturbance–recovery context where community assembly processes (succession) are central.

Connectivity is a key element of recovery dynamics, influencing the supply of colonists and defining the relative importance of regional- and local-scale processes [15]. Disturbance and recovery processes can create a landscape of patches at different stages of recovery, influencing the maintenance of diversity in complex community networks and the potential for cumulative effects to occur through repeated small-scale disturbance events. Metacommunity models emphasize the importance of the interaction of connectivity between patches and local community dynamics (within patches) in driving change at multiple spatial scales [16]. This underlying theory can allow field studies and manipulative experiments to address recovery and resilience within the context of habitat loss, connectivity and community homogenization [1,10,11,17].

The plant and animal communities of temperate rocky shores exhibit a wide range of dispersal strategies, despite the continued perception of marine communities as ‘open'. Recruitment to disturbed sites can occur through larval or propagule dispersal and movement of juvenile or adult life stages. The importance of different modes of colonization will depend on the natural history of species, interactions with environmental conditions (particularly hydrodynamics) and space and time scales. The complexity of dispersal mechanisms highlights the difficulty of measuring and modelling dispersal at the community scale and the value of finding surrogate measures of connectivity based on natural history, landscape connectivity and community ecology. β-diversity can be generated by multiple processes associated with metacommunities, (e.g. stochastic processes or environmental filtering) but dispersal plays a role in affecting the relative importance of any process. β-diversity has been proposed as a surrogate for whole community dispersal as it encompasses the relationship between site species richness and the regional species pool [1,11].

While many experiments on rocky shores have investigated recovery dynamics, they have often focused on small disturbance patches (usually 20 cm diameter), which may overemphasize the role of local recovery processes. Moreover, usually the community investigated consists only of dominant epibenthos (macroalgae, large suspension feeders, mobile grazers and predators). However, living in the interstices between these visible epibenthos are many small invertebrates that contribute to species richness [18]. In a novel manipulative experiment, we completely cleared large patches (1 m^2^) that encompassed the entire tidal range; these plots were replicated across six sites encompassing 80 km of shoreline. The experimental design facilitated multi-scale inference across sites and within sites, in order to assess differences in the relative rate of community recovery and the relative importance of local and regional processes in determining recovery rate. Specifically, we tested the role of local (within-site) and regional variables (varying between sites) in the relative recovery of two components of the benthic community: epibenthos (which were dominated by macroalgae and mussels (i.e. habitat structure formers)) and infauna (small animals living in the macroalgal assemblage, turf or canopy). Two scales of β-diversity were used as surrogates: ecological homogeneity, within-site β-diversity (i.e. β-diversity is inversely correlated with heterogeneity); and ecological connectivity, among-site β-diversity (i.e. low β means well connected). This surrogate of ecological connectivity reflects both hydrodynamic constraints and life-history traits for all the species sampled at a particular site. We also estimated the nestedness, replacement and richness difference components of β-diversity to provide insight into the relative importance of habitat filtering and niche-related processes (replacement) and stochastic processes (nestedness) in undisturbed and disturbed plots [19]. We note that, essentially, replacement and nestedness are inverse components of β-diversity and are influenced by the heterogeneity of the landscape, spatial scale and dispersal. While the relative contributions of different β-diversity components may be clear-cut in theory, in empirical research any aspect of diversity is probably the outcome of multiple community assembly processes [12,19–21].

We predicted that (1) the relative contributions of different components of β-diversity would differ between undisturbed and experimentally disturbed communities, with disturbed communities more likely to exhibit replacement. In our experimental study, we expected nestedness to dominate the undisturbed community because our samples of the community integrate over an extended time period, allowing stochastic processes to dominate β-diversity [22]. In the recovering disturbed plots, we expected species replacement owing to both spatial and temporal factors associated with plot succession to dominate β-diversity [23]. However, the relative importance of nestedness and replacement may vary with spatial scale, hence prediction (2): sites closely connected to the regional species pool (low among-site β-diversity) would recover faster, particularly if replacement dominated plot recovery (prediction 1), as we expected that strong environmental filters (indicated by the high-nestedness component of β-diversity) would slow relative recovery. Prediction (3): biogenic habitat creation will lead to stronger habitat filtering of infauna than epibenthos, as macroalgae and mussels facilitate infaunal recovery. Therefore, relationships between β-diversity and recovery would differ for epibenthos and infauna.

## 2. Methods

### 2.1. Study sites

Six experimental sites were distributed over 80 km of the eastern Ligurian coast (northwestern Mediterranean), encompassing the two Marine Protected Areas (MPAs) of Portofino and Cinque Terre (electronic supplementary material, figure S1 and table S1): one site near Genoa city (Pontetto (PON)), one in the C zone of the Portofino MPA (Punta Chiappa (POR)), one close to Framura (FRA), one close to Bonassola (BON) and two in the A zone of the Cinque Terre MPA (Punta Mesco (MES) and Montenero (MON)). The tidal range in this region is narrow (around 30 cm) and the barometric tide (related to prolonged atmospheric high-pressure conditions, particularly in the summer) may dominate the water level. All sites had similar wave exposure and wind-driven flows are the major hydrodynamic forcing in this area. Sites were also similar in terms of shore slope, but showed differences in terms of the dominant macroalgal assemblage. In particular, the local macroalgal community in PON was dominated by canopy-forming species of the genus *Cystoseira*, followed by *Corallina elongata* (articulated Corallinales); in POR the dominant taxa were articulated Corallinales and fleshy red *Laurencia* spp.; in FRA and BON the assemblage was dominated by *Cystoseira compressa*, articulated and encrusting Corallinales and turf-forming species (made up of a complex and indiscernible matrix of small Corallinales, Ceramiales and other filamentous algae); in PES and MON algal turf and articulated Corallinales were dominant. Additional information on site features is reported in [18] (see also the electronic supplementary material).

### 2.2. Experimental design and sampling

Across the intertidal area of each site, five disturbed plots (1 m × 1 m), each separated by about 10 m, were established in May 2009. Each disturbed plot was completely cleared of biota by a combination of high-pressure water and sand blasting (at 160 bar). Sampling for recovery of large immobile visually detectable organisms (mussels, barnacles but predominantly macroalgae, henceforth called epibenthos) and infauna occurred after 1 year, in July 2010. Epibenthos per cent cover (see [24]) was estimated in a 20 × 20 cm quadrat located at the centre of the disturbed plot. Organisms were classified at the lowest taxonomic resolution possible and data reported as per cent cover. Undisturbed quadrats were paired with each disturbed plot and collected 0.5 m away from the plot perimeter and at the same elevations of the disturbed samples. The infauna (the fauna living in the macroalgal assemblage, turf or canopy) was collected with a corer (5 cm internal diameter), at a single position haphazardly located within each disturbed and paired undisturbed plot. All samples were preserved with 70% isopropyl alcohol and stained with Rose Bengal; organisms (mostly polychaetes, bivalves and amphipods) were enumerated at the lowest taxonomic resolution possible. Data were reported as individuals per centimetre square.

### 2.3. Site environmental characteristics

To characterize differences within and between sites, the following indices were calculated.

(i) Habitat complexity in terms of sinuosity of the shoreline was measured at different scales using the following indices: (a) sinuosity (chain), within site, by way of carefully laying a chain (oval links of internal diameter 1.4 × 6 mm; chain length 11.4 m) along the coast following minor indentations at the same tidal height as our disturbed plots (five measurements per site); (b) two different measures of fractal dimension were used to provide measurements of habitat rugosity at the larger scale of the whole site extent (150 m); both these indices were calculated using ARCGIS. Fractal (GIS) was defined as: *D* = log(*n*)/[log(*n*) + log(*d*/*L*)], where *n* is the total number of line segments, *d* is the distance between the start and endpoints of the line, and *L* is the cumulative length of all line segments (i.e. the individual segments of the continuous broken line of the coastline), where *d* was set at the highest level of resolution on the 1 : 5000 map of the Ligurian coastline. Sinuosity (GIS) was defined as: *S* = *l*/*l*_sf_; where *l* is the cumulative length of all line segments, and *l*_sf_ is the distance between the start and endpoints of the transect. Sinuosity (GIS) is calculated in the same way as sinuosity (chain) measurements, but at a larger scale: the sinuosity (chain) taking into account the site-scale roughness of the substrate.
(ii) Given the different complexity of the dominant macroalgal assemblages, an algal habitat complexity index (AHCI), indicating three-dimensionality and shelter provision, was calculated (see [18]), based on plant morphology and their per cent cover. The AHCI was calculated at the site level, allocating 30 random 20 × 20 cm quadrats. For each quadrat, the morphology of individual species was allocated to 1 of 5 groups. These ranged from coralline crusts and algal films (score 1) to filamentous turf (score 2), coralline turf (score 4), small bushy algae (score 7) and large ‘canopy' formers (score 10; note the largest algae on these shores are only approx. 20 cm tall). The scores of the five groups were multiplied by their per cent cover and summed to provide the index.
(iii) An urbanization index for each site was calculated by dividing the population density of the closest urban centre (ind km^−2^, www.comuni-italiani.it) by the shortest distance (m) to the site.
(iv) A river index was calculated as the river flow (dm^3^ sec^−1^) of the closest river divided by the distance (m) from the sampling site. This simple index is a good surrogate for river effects because of the weak near shore currents and the dominance of weather conditions on water movement.
(v) An exposure index was calculated as the aspect of each site relative to the main wave direction (225°).

### 2.4. Site ecological characteristics

(i) Relative recovery of epibenthos and infauna for each plot was estimated as the difference between paired disturbed and undisturbed samples 1 year after disturbance, based on Bray-Curtis dissimilarities of raw count data as percentages (i.e. community recovery = 100 - % Bray-Curtis dissimilarity).
(ii) We calculated β-diversity, based on presence–absence data, at two scales, within and among sites, based on undisturbed data only [25]. Within-site β-diversity was measured as the difference between the average number of species found at each site and the total number of species found at the site. Among-site β-diversity was calculated as the difference between the average number of species found at each site (average α-diversity) and the total number of species found across all sites (region γ-diversity). Given the overall similar levels of richness across sites, we did not consider it necessary to control for variation in species richness in calculating β-diversity as advocated by [26]; but see also [27–29]. Note that we also trialled the use of the local contribution to β-diversity index [12]; however, this did not prove to be useful in this context.
(iii) We decomposed the undisturbed β-diversity into replacement, richness difference and nestedness, based on the Jaccard group (presence/absence data) and using the Podani family of calculations [12,13]:
relativized species replacement (*R*_repl_) = 2 × min (*b*, *c*)/ (*a* + *b* + *c*),
relativized richness difference (*R*_rd_) = |*b* − *c*|/(*a* + *b* + *c*),
relativized nestedness (*R*_nest_) = (*a* + |*b* − *c*|)/(*a* + *b* + *c*), if *a* > 0 and 0 if *a* = 0, which equates to 1–*R*_repl_,
where for each *i*,*j* pairwise comparison, *a* = number of taxa in common, *b* = number of taxa only observed in sample*_i_*, *c* = number of taxa only observed in sample*_j_*.
Calculations were performed in Excel, but they may also be computed using the beta.div.comp function in the R software, of the adespatial package.
To estimate the contribution of replacement, nestedness or richness difference among the sites within the study area, and of each plot among the plots of each site, calculations were done on two scales. From the 15 pairwise comparisons among sites, and the 10 pairwise comparisons for each plot at the within-site scale, we calculated the average of the replacement and richness difference components for each site and plot to be used in testing predictions 2 and 3.
(iv) On disturbed plot data we calculated the replacement, nested and richness difference at the among-site scale only.

### 2.5. Statistical analyses

#### 2.5.1. General experimental results

An initial PERMANOVA analysis was performed on epibenthic and infaunal composition data separately, to test for treatment and site effects (treatment, fixed, two levels, crossed; site, random, six levels, crossed; Primer 6 and PERMANOVA + β3 software).

#### 2.5.2. Prediction 1

Differences in the relative contribution of different components of β-diversity between undisturbed and experimentally disturbed communities were tested by paired *t*-tests for each component separately. This was done for the among-site scale only (*n* = 15).

#### 2.5.3. Predictions 2 and 3

Multiple regression was used to develop the best predictive model for each assemblage type. All environmental and β-diversity variables described above were used in the modelling, with nonlinearities incorporated by using log transformations, polynomials and interactions. We expected differences in relative recovery to be influenced by both within-site and between-site processes. While some variables were only available at the larger scale (among sites), other variables (e.g. recovery, within-site β-diversity, average plot replacement and richness difference) differed at the plot level within sites, resulting in an *n* of 30 for the regression analyses. Parsimonious models were produced by backwards selection based on *p*-values, with terms only removed if doing so did not result in a significant increase in deviance [30]. The problem of correlations between predictor variables was dealt with by selecting one of the correlated variables to use in the model, finding the most parsimonious model and then beginning the process again using the other variable instead. The best of these models was selected based on residual by predicted plots, residual normal plots, partial leverage plots, stability of the parameter estimates and the Akaike's information criterion (AIC) [31–33]. Over fitting was controlled for by (a) restricting polynomials to second order and (b) examining the retention of predictor variables within the final models by the AIC, the Bayes information criteria (BIC) and Mallows' C*_p_* statistic. None of the final models retained variables with a variance inflation factor greater than 5, nor did they include polynomials.

## 3. Results

### 3.1. General experimental results

One year after clearing the 1 m^2^ plots, recovery was not complete for either infauna or epibenthos, with the average recovery per site ranging from 30 to 60 for infauna and 15 to 59 for epibenthos). For the infauna there was no significant site*treatment interaction, but a significant treatment effect (table 1). Epibenthos showed a significant site*treatment interaction, with the treatment effect, while always significant, varying in strength across sites. BON and POR exhibited the largest difference between undisturbed and treatment (indicating slowest recovery, *p* = 0.007 and *p* = 0.008), while FRA exhibited the fastest recovery (*p* = 0.029). The other three sites also displayed a significant treatment effect (PON: *p* = 0.011; PES: *p* = 0.012; and MON: *p* = 0.016).

**Table 1.** Significance of differences in community structure between disturbed and undisturbed plots 1 year after disturbance, for the epibenthic and the infaunal components of the community.

		epibenthos			infauna		
source	d.f.	MS	pseudo-F	*p*-value	MS	pseudo-F	*p*-value
site	5	6326.4	6.64	0.001	3624.9	1.97	0.007
treatment	1	22078	9.11	0.001	16184	9.15	0.001
site × treatment	5	2431.3	2.55	0.001	1768.2	0.961	0.524
residuals	46	952.25			1837.4		
total	57						

### 3.2. Prediction 1

**Figure 1.**
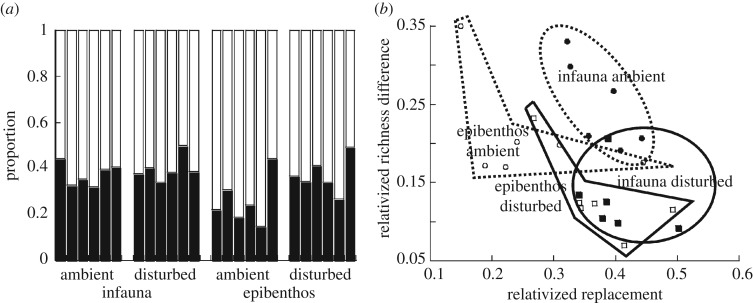
Relative contributions of nestedness, replacement and richness difference to β-diversity, calculated as per the Jaccard group (presence/absence) from the Podani family. (*a*) Relative proportions of average site replacement (bottom) and nestedness (top). (*b*) Relationship of average site richness difference to average site replacement for infauna (infilled symbols) and epibenthos (open symbols) where undisturbed values are circles and disturbed values are squares.

Across sites, epibenthic relativized replacement was higher for disturbed plots than undisturbed ones (paired *t*-test, *n* = 15, *p* = 0.0004; figure 1*a*), prediction 1, and conversely relativized nestedness and richness difference were higher in undisturbed plots than in disturbed ones (paired *t*-test, *n* = 15, *p* = 0.0004 and *p* < 0.0001, respectively). Results were not so clear for the infaunal β-diversity components, with significant effects only observed for relativized richness difference (paired *t*-tests, *n* = 15, *p* = 0.0240, undisturbed > disturbed). Overall, the sites ranked based on nestedness were different for infauna and epibenthos (figure 1*a*). A significant effect of infauna and epibenthos was not detected on the relativized richness difference proportion of β-diversity for either undisturbed or disturbed plots (*p* > 0.20 for both; figure 1*b*). Epibenthic and infaunal communities from both the undisturbed and the disturbed plots demonstrated higher proportions of relativized nestedness than replacement (paired *t*-tests, *n* = 15, *p* < 0.01 for all four sets of comparisons).

### 3.3. Predictions 2 and 3

The best model for epibenthic recovery included a negative relationship for within-site β-diversity, and a positive relationship with control plot number of taxa (*R*^2^ = 0.64; *p* < 0.0001; table 2). Much more complicated results were found for the recovery of the infaunal community. The model suggested by the AIC statistic included six predictor variables, two as squared terms and two involved in a multiplicative interaction (AIC = 133.19, BIC = 139.64, C_*p*_ = 7, *R*^2^ = 0.60, *p* = 0.0020; table 2). Infaunal recovery was positively related to epibenthic recovery in the plot (squared), with this effect moderated by the undisturbed per cent cover of coralline algae (squared). Recovery was also negatively affected by average plot relativized richness difference and average plot relativized replacement. Finally, at the among-site scale, there was a negative effect of average site relativized replacement and a positive effect of average site relativized replacement multiplied by among-site β-diversity.

**Table 2.** Statistical information of the final model predicting differences in the relative recovery of epibenthos and infauna between sites, 1 year after defaunation.

variable	*R*^2^	d.f.	estimate	*F*-value	*p*-value
epibenthos					
model	0.64	2		22.18	<0.0001
error		25			
control plot number of taxa		1	2.80	1.60	0.1228
within-site β-diversity		1	−6.63	−6.23	<0.0001
infauna					
model	0.60	6		5.23	0.0020
error		21			
(epibenthic recovery)^2^		1	0.0032	2.36	0.0283
(Corallina cover)^2^		1	−1.22	−3.23	0.0040
average site replacement * infaunal among-site β		1	18.72	2.48	0.0215
average plot replacement		1	−61.28	2.29	0.0326
average plot richness difference		1	−73.37	−2.27	0.0335
average site replacement		1	−394.24	−2.11	0.0467

## 4. Discussion

Prediction 1 (that undisturbed communities would exhibit higher nestedness and disturbed communities would exhibit higher replacement) was only observed for the epibenthic communities. Our models showed no indications that sites with higher connectivity to the regional species pool recovered faster for epibenthos or infauna (prediction 2). Our third prediction (that relationships between recovery and β-diversity would differ for epibenthos and infauna) was the most strongly supported of our three predictions.

Environmental factors were not important either for epibenthic or infaunal recovery. Instead, our results emphasize the importance of local biotic features in affecting the relative recovery rates of rocky shore communities. Epibenthic (mainly macroalgal) recovery was faster at homogeneous sites (i.e. sites characterized by low within-site β-diversity) and where high numbers of taxa were found in the nearby undisturbed plots. Similarly, infaunal recovery increased with increased recovery of the epibenthos (prediction 3), negatively mediated by local habitat structure (coralline algae). However, for infaunal recovery, effects of local habitat were overlain by aspects of β-diversity at both the local and among-site scale.

For macroalgae, limited macroalgal propagule dispersal and/or colonization through vegetative propagating thalli contribute to the domination of local over regional processes in recovery. Vegetative propagating thalli appear to be less vulnerable to a variety of physical and biological factors (e.g. wave action, sediment stress, bottom instability, herbivory, competition) than are sexual propagules, typical of canopy-forming species [34]. Thus, vegetative propagation should be an important mechanism of recovery after most common disturbances, especially when damage to algae is patchy [35]. In fact, algal turf-dominated communities (such as FRA) characterized by vegetative propagation, recovered much faster than canopy-dominated ones (mostly PON, POR and BON; [24,36], which rely on recolonization by sexual propagules.

The model for the infauna emphasizes the importance of recovering macroalgae influencing differences in recovery. Interestingly, despite the importance of macroalgae in habitat creation for infauna, the recovery of these communities did not simply track habitat formation (per cent explained less than 45%), with other processes also indicated as important (e.g. average plot richness difference, average plot replacement and average site replacement of infaunal communities). In particular recovery was increased when both average site replacement and among-site β-diversity were high. While previous surveys of macroalgae and associated infauna have emphasized the importance of site α-diversity and the habitat architecture created by different algal morphologies [18], our results suggest that documenting changes in the large plants and animals alone will be a poor surrogate for rocky shore community assembly processes.

Using β-diversity as an indicator of ecological connectivity in this large-scale experiment indicated no reliance on the regional species pool for epibenthic or infaunal recovery, thus decreasing the risk of cumulative impacts propagating along the coast. Conversely, this implies limited resilience at the site scale (10–100 m) to large-scale disturbance associated with impacts such as landslides, oil spills or shoreline modification.

Our results can be scaled up to the regional scale because we employed an unusually large scale of disturbance encompassing much of the entire tidal range in the Mediterranean and we replicated our experiment across a range of sites that encompassed major rocky shore habitat variation in the region. We have much to learn about resilience, connectivity and thresholds of recovery from unfavourable states. Mixing experimental studies of disturbance and recovery with information on different scales of heterogeneity in biodiversity allows us to gather information on a range of scales and ecological processes. In this system, we found that local ecological features associated with β-diversity and biogenic habitat formation influenced community assembly through habitat filtering and species replacement (turnover) in disturbed plots. An important implication of this is our ability to interpret survey data when the disturbance history is unknown [4,37]. Natural landscapes often represent a mosaic of patches in different stages of recovery: if ignored, these may confuse the analysis of patterns in biodiversity, as both the spatial scale of patchiness and the temporal scale of disturbance history may be expected to alter the relative contributions of replacement and nestedness. However, future research may reveal the value of assessing different components of β-diversity in identifying the position of a specific site's community to a critical transition zone.

Connectivity is the consequence of interactions between natural history, transport vectors and the quantity, quality and spatial arrangement of habitat patches, across landscapes. As such, integrative measures or surrogates for connectivity are likely to be imperfect. Nevertheless, as disturbance to coastal habitats increases and biodiversity continues to be eroded, connectivity and the current state of ecological communities will play an important role in understanding the consequences of change in different ecosystems. Increasing the disturbance regime ultimately leads to the homogenization of habitats and communities. These effects are likely to occur faster as the spatial scale of disturbance increases [38]. Our finding that more homogeneous sites recover faster suggests that the increase in recovery potential of simplified communities speeds the synergic interplay of fragmentation and decrease in community complexity and biodiversity.

## Supplementary Material

Table S1 from The multiple roles of β–diversity help untangle community assembly processes affecting recovery of temperate rocky shores

## Data accessibility

Data are available at Dryad (http://dx.doi.org/10.5061/dryad.rs467t5) [39].
