# Supplementary material for: The multiple roles of β-diversity help untangle community assembly processes affecting recovery of temperate rocky shores
Source: R Soc Open Sci. 2018 Aug 8;5(8):171700. doi: 10.1098/rsos.171700 (PMC6124088; doi:10.1098/rsos.171700)
Supplement: Table S1 from The multiple roles of β–diversity help untangle community assembly processes affecting recovery of temperate rocky shores [file rsos171700supp1.docx]

SUPPLEMENTARY MATERIAL - A

FOR

The multiple roles of β–diversity help untangle community assembly processes affecting recovery of temperate rocky shores

BY

Mariachiara Chiantore, Simon F Thrush, Valentina Asnaghi, Judi E Hewitt

Figure S1: Location of sites along Liguria coastline





Table S1: Location and environmental data for experimental sites

| Location | Lat °N | Long °E | Chain | Fractal  dimension | Sinuosity | Exposure  from 225° | Urbanization  index | River  Index | Algal Habitat Complexity Index |
| --- | --- | --- | --- | --- | --- | --- | --- | --- | --- |
| PON | 44.3756 | 9.0757 | 8.29 | 1.182 | 1.672 | 0.91 | 1.331 | 0.217 | 582.0 |
| POR | 44.3230 | 9.1460 | 7.58 | 1.026 | 1.083 | 0.09 | 0.195 | 0.250 | 450.5 |
| FRA | 44.1993 | 9.5560 | 8 | 1.052 | 1.183 | 0.97 | 0.035 | 1.036 | 280.3 |
| BON | 44.1807 | 9.5751 | 9.39 | 1.035 | 1.116 | 0.09 | 0.114 | 1.102 | 332.5 |
| MES | 44.1392 | 9.6279 | 7.9 | 1.010 | 1.030 | 0.26 | 0.065 | 1.342 | 514.9 |
| MON | 44.0930 | 9.7384 | 8.62 | 1.022 | 1.080 | 0.97 | 0.221 | 1.923 | 502.9 |

Table s2: Taxa observed in epibenthic and infaunal sampling in July 2010

| **Infaunal taxa** | **Infaunal taxa (continued)** | **Epibenthos** |
| --- | --- | --- |
| Elasmopus pocillimanus | Patella sp | Acetabularia acetabulatum |
| Stenothoe tergestina | Mytilus galloprovincialis | Bryozoans |
| Podolerus variegatus | Musculus costulatus | Cirripedi |
| Caprella equilibra | Perinereis cultrifera | Cladophora |
| Caprella grandimana | Platynereis dumerilii | Colpomenia sp |
| Caprella hirsuta | Syllis gracilis | Corallina elongata |
| Leptocherius pectinatus | Syllis prolifera | Cystoseira amentacea var stricta |
| Jassa marmorata | Syllis sp | Cystoseira compressa |
| Sphaeromatidae | Nereis jacksoni | Dictyotales |
| Anthuridea | Sabellidae | Encrusting algae Non Corallinales |
| Tanais dulongii | Capitellidae | Gastroclonium clavatum |
| Leptocheila saviceyi | Rhynchothorax voxorinum | Hypnea musciformis |
| Harpacticoid | Anoplodactylus petiolatus | Jania rubens |
| Balanus sp | Nematodi indet | Laurencia complex |
| Granchio indet |  | Mytilus galloprovincialis |
| Acanthochitona fascicolata |  | Padina pavonica |
| Gastrochaena dubia |  | Algal turf complex |

Figure S2: Predicted response curves for the epibenthos recovery model





Figure S2: Predicted response curves for the infaunal recovery model
